# Substituting partial chemical nitrogen fertilizers with organic fertilizers maintains grain yield and increases nitrogen use efficiency in maize

**DOI:** 10.3389/fpls.2024.1442123

**Published:** 2024-09-18

**Authors:** Le Wang, Hongliang Zhou, Cong Fei

**Affiliations:** ^1^ State Key Laboratory of Aridland Crop Science, Agronomy College, Gansu Agricultural University, Lanzhou, Gansu, China; ^2^ Agronomy College, Shihezi University, Shihezi, Xinjiang, China; ^3^ Department of Life Sciences, Yuncheng University, Yuncheng, Shanxi, China

**Keywords:** organic fertilizers, rainfed maize, nitrogen recovery efficiency, grain yield, semi-arid region

## Abstract

**Introduction:**

Long-term application of excessive nitrogen (N) not only leads to low N use efficiency (NUE) but also exacerbates the risk of environmental pollution due to N losses. Substituting partial chemical N with organic fertilizer (SP) is an environmentally friendly and sustainable fertilization practice. However, the appropriate rate of SP in rainfed maize cropping systems in semi-arid regions of China is unknown.

**Methods:**

Therefore, we conducted a field experiment between 2021 and 2022 in a semi-arid region of Northern China to investigate the effects of SP on maize growth, carbon and N metabolism (C/NM), and NUE. The following treatments were used in the experiment: no N application (CK), 100% chemical N (SP0, 210 kg N ha^–1^), and SP substituting 15% (SP1), 30% (SP2), 45% (SP3), and 60% (SP4) of the chemical N. The relationship between these indicators and grain yield (GY) was explored using the Mantel test and structural equation modeling (SEM).

**Results and discussion:**

The results found that the SP1 and SP2 treatments improved the assimilates production capacity of the canopy by increasing the leaf area index, total chlorophyll content, and net photosynthetic rate, improving dry matter accumulation (DMA) by 6.2%–10.6%, compared to the SP0 treatment. SP1 and SP2 treatments increased total soluble sugars, starch, free amino acids, and soluble protein contents in ear leaves via increasing the enzymatic reactions related to C/NM in ear leaves during the reproductive growth stage compared with SP0 treatment. The highest plant nitrogen uptake (PNU) and nitrogen recovery efficiency were obtained under the SP2 treatment, and the GY and nitrogen agronomic efficiency were higher than the SP0 treatment by 9.2% and 27.8%. However, SP3 and SP4 treatments reduced DMA and GY by inhibiting C/NM in ear leaves compared to SP0 treatment. Mantel test and SEM results revealed that SP treatments indirectly increased GY and PNU by directly positively regulating C/NM in maize ear leaves. Therefore, in the semi-arid regions, substituting 30% of the chemical N with SP could be considered. This fertilizer regime may avoid GY reduction and improve NUE. This study provides new insights into sustainable cultivation pathways for maize in semi-arid regions.

## 1 Introduction

The exponential surge in food demand due to the rapid growth in the population globally and the reduction in crop yields by extreme global climate change poses significant challenges to agricultural production. Maize is a vital food crop with wide adaptability, high productivity, and versatility. In China, maize cultivation spanned 44.22 million hectares in 2023, widely distributed across all provinces (National Bureau of Statistics, 2023). In recent decades, Chinese maize growers have boosted maize yields using large amounts of chemical fertilizers. However, prolonged and excessive use of chemical fertilizers resulted in environmental problems, including soil quality degradation and agricultural pollution (Zhang et al., 2021; He et al., 2022a; He et al., 2022b; Wang et al., 2023). Overuse of chemical fertilizers increases the cost of agricultural production and potentially hampers crop growth, reducing maize yield and quality (Liu L. et al., 2022; Yan et al., 2023). China has been implementing measures to reduce fertilizer use since 2015 and achieve zero growth in fertilizer application by 2025 (He et al., 2022a; Cao et al., 2023). Consequently, exploring practices that ensure sustainable, stable, and high maize yields is critical while reducing chemical fertilizer application.

With the increasing awareness of environmental protection and food safety in people, organic fertilizers (SP), green and environmentally friendly fertilizer resources, are becoming popular. Replacing partial chemical nitrogen (N) fertilizers with SP is more conducive to achieving stable and high crop yields (Burke et al., 2022; He et al., 2022b). SP mainly includes animal manure, crop residues, and industrial and urban wastes. They are rich in organic matter (such as amino acids, organic acids, and peptides) and inorganic nutrients like N, phosphorus (P), and potassium (K). These fertilizers improve soil structure and fertility and enhance plant resistance to disease (Zhang et al., 2021; Zhai et al., 2022). SP provides nutrients for crop growth, improves the physical and chemical properties of the soil by increasing the soil’s organic carbon content and active and inert carbon content, and enhances the soil’s water and fertilizer retention capacity, thereby increasing crop water use efficiency (Wang et al., 2022a; Wu G. et al., 2024). However, nutrient release from SP is slow, and the release rate is influenced by soil temperature and moisture. This slow release may fail to ensure sufficient nutrient supply during rapid crop growth or yield formation (Liang et al., 2023). Therefore, to meet nutrient requirements during crop growth and yield formation, the excessive substitution of SP should be avoided; otherwise, it may result in yield reductions (Liu Z. et al., 2022; He et al., 2022a). Moreover, the appropriate substitution ratios should be adjusted according to the climatic and soil conditions of the region (Bhunia et al., 2021; Wu G. et al., 2024). Many researchers demonstrated the effects of SP on maize growth and yield (Zhai et al., 2022; Fudjoe et al., 2022); however, previous research only focused on the potential of SP in improving soil physicochemical properties (Zhang et al., 2021; He et al., 2022a; He et al., 2022b), and microbial community structure (Li et al., 2023; Hu W. et al., 2023) and reducing greenhouse gas emissions (Wang et al., 2020; Hu Y. et al., 2023; Hu W. et al., 2023). A few studies investigated how SP affects yield formation by regulating physiological and metabolic processes in maize plants.

Carbon and N metabolism (C/NM) are crucial physiological metabolic pathways in crops, essential for crop growth and yield formation (Hu et al., 2021); leaf C/NM directly affects crop growth and yield formation (Wang et al., 2023; Li et al., 2023). Carbon metabolism mainly involves carbon assimilation, reduction, and transformation in photosynthesis, providing energy and structural materials for plants. N metabolism involves N uptake, assimilation, and conversion, which provide the basis for synthesizing vital substances such as proteins and nucleic acids (Liang et al., 2023; Liu et al., 2024). N metabolism requires carbon metabolism to provide a carbon skeleton and energy support, while carbon metabolism needs nitrogen metabolism to provide the involvement of enzymes and photosynthetic pigments, among other substances (Erdal, 2019). Maintaining high levels of leaf C/NM in maize during the reproductive growth stage using agronomic practices improves grain yield (GY) and resource use efficiency (Liang et al., 2023; Li et al., 2023, 2024). SP can balance the nutrient requirements of maize during different growth stages by regulating the availability of fast-acting nutrients in the soil, demonstrating the potential of SP in enhancing the maize leaf C/NM (Wang et al., 2023; Liang et al., 2023; Zhai et al., 2024). However, it remains unclear which SP ratios can maintain high levels of leaf C/NM processes during reproductive growth in rainfed maize cropping systems in semi-arid regions.

We investigated the effects of SP on maize plant growth, photosynthesis, C/NM, and GY by setting up different SP substitution ratios in a rainfed maize cropping system in a semi-arid region of Northern China. We hypothesized that (1) an appropriate substitution ratio of SP promotes assimilate production and plant growth by increasing photosynthetic area, pigment content, and gas exchange capacity. (2) An appropriate substitution ratio of SP enhances the transfer of carbon and N metabolites to the kernel by accelerating enzymatic reactions related to C/NM in leaves during the reproductive growth stage, ultimately increasing GY. This study aimed to reveal the mechanism by which SP stabilizes and increases maize yield and provide novel insights into sustainable production pathways that reduce fertilizer dependence and improve resource use efficiency in rainfed maize cropping systems in semi-arid regions.

## 2 Materials and methods

### 2.1 Experimental site

**Figure 1:**
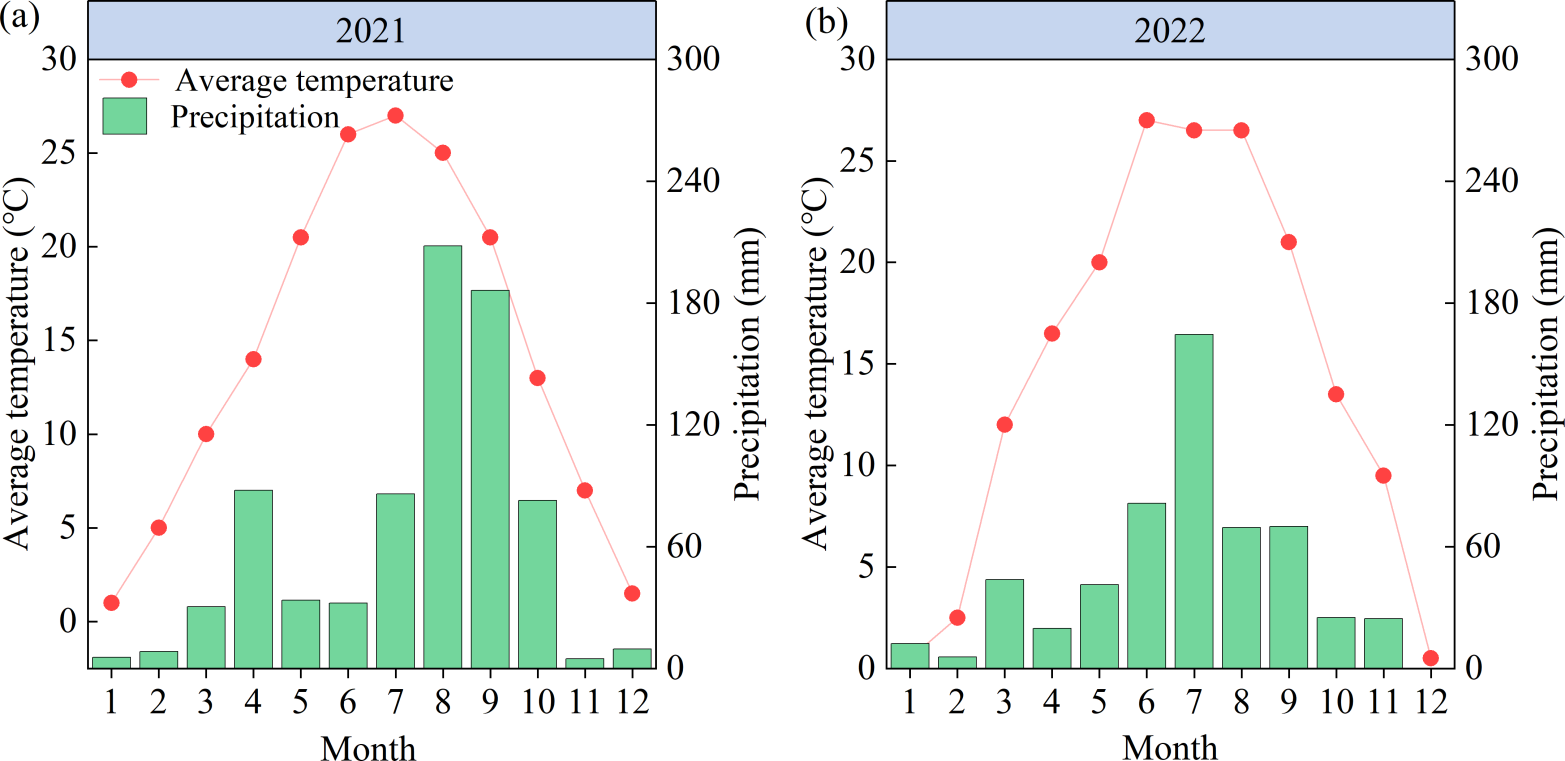
**(A, B)** Average air temperature and precipitation in the experiment region in 2021 and 2022.

The experiment was conducted in 2021 and 2022 in Xiedian Town, Wanrong County, Shanxi Province (110°86′E, 35°44′N). The experimental region was situated in a temperate continental monsoon climate zone at an elevation of 354.5 m. The average temperature over the years was 14.4°C, the average annual precipitation was 624.5 mm, and the average total sunshine hours was 2216 h (Wang et al., 2022b). Monthly precipitation and average temperature for 2021 and 2022 are given in **Figure 1**; the precipitation for the two maize growing seasons (May–September) was 515.9 and 426.2 mm, respectively. The soil type is brown loamy soil with a light loamy texture. The previous crop was wheat, and soil samples were collected from the 0–20 cm soil layer of the test site before sowing. The soil physicochemical properties were determined according to the method reported by Bao (2000). The soil organic matter content before sowing in both years was 23.5 g kg^–1^, total N 1.12 g kg^–1^, hydrolyzable N 113.4 mg kg^–1^, total P 0.85 g kg^–1^, available P 25.52 mg kg^–1^, available K 158.2 mg kg^–1^, and pH 7.9.

### 2.2 Experimental design

A randomized block design was employed, including no N fertilization (0 kg N ha^–1^) (CK) treatment and five substituting partial chemical N fertilizers with SP treatments (**Table 1**). The five treatments were 0% chemical N fertilizer substitution with SP (SP0, 210 kg N ha^–1^), 15% chemical N fertilizer substitution with SP (SP1), 30% chemical N fertilizer substitution with SP (SP2), 45% chemical N fertilizer substitution with SP (SP3), and 60% chemical N fertilizer substitution with SP (SP4). There were three replications for each treatment, totaling 18 plots, each with an area of 50 m^2^ (10 × 5 m). The maize variety was ‘Denghai 605’ (Denghai Seed Industry Co., Ltd, Shandong, China), which is widely grown in the region. The seeds were sown on May 5, 2021, and May 7, 2022, at a planting density of 60,000 plant ha^–1^, with a row spacing of 55 cm and a plant spacing of 30 cm.

**Table 1:** Application rates of chemical nitrogen and organic fertilizers, calcium superphosphate, and potassium sulfate in different treatments.

Treatments	Urea kg ha^–1^	Organic fertilizer kg ha^–1^	Calcium super phosphate kg ha^–1^	Potassium sulfate kg ha^–1^	Total N kg ha^–1^	Total P_2_O kg ha^–1^	Total K_2_O kg ha^–1^
CK	0	0	750	300	0	120	150
SP0	457	0	750	300	210	120	150
SP1	388	1050	684	279	210	120	150
SP2	320	2100	619	258	210	120	150
SP3	251	3150	553	237	210	120	150
SP4	183	4200	488	216	210	120	150

CK refers to no chemical and organic nitrogen fertilizer. SP0 refers to 100% chemical nitrogen fertilizer (210 kg N·ha^–1^), and SP1, SP2, SP3, and SP4 refer to 15%, 30%, 45%, and 60% of chemical nitrogen fertilizer substituted with organic fertilizer, respectively.

The chemical N fertilizer was urea (46% N content), and the SP was granular SP produced by the fermentation of chicken manure (Yinghe Biological Technology Co., Ltd, Shijiazhuang, China). The organic fertilizer contained 45% organic matter, 3% total N, 1.0% total P, and 1.0% total K. The total N application rate for all treatments was 210 kg N ha^–1^, and the details of SP substituted chemical N are provided in **Table 1**. The applied SP contained small amounts of P and K. To ensure consistent application rates of P fertilizers (120 kg ha^–1^) and potash fertilizers (150 kg ha^–1^) across all treatments, the shortages were supplemented using calcium superphosphate (16% of P_2_O_5_ content) and potassium sulfate (50% of K_2_O content). Before sowing, all organic, P, and potash fertilizers were applied as basal fertilizers and thoroughly mixed with the soil using a tractor (Zhongjin Construction Machinery Co., Ltd, Jining, China). Moreover, 40% of the chemical N fertilizer was used as basal fertilizer, and 60% was applied manually in holes at the bell-mouth stage (twelve-leaf stage, V12). All plots received the same agronomic management, including weeding and application of insecticides.

### 2.3 Gas exchange and photosynthetic enzyme activity

At the six-leaf (jointing stage, V6), twelve-leaf (bell-mouth stage, V12), silking (R1), and milk maturity (R3) stages in the two growing seasons, three uniformly growing plants were selected from each plot. The net photosynthesis (Pn) of the ear leaves was measured using the LI-6400 portable photosynthesis system (LI-COR, Biosciences, Lincoln, NE, USA) from 10 a.m. to 12 p.m. on a sunny day. For Pn measurement, the light intensity was set at 1600 µmol m^–2^ s^–1^ using LED red and blue light sources, the CO_2_ concentration was set at 400 µmol CO_2_ mol^–1^ (provided by CO_2_ gas cylinders), and the temperature in the leaf chamber was set at 25°C. After measuring the gas exchange, a part of the ear leaves was cut and stored in an ultra-low temperature freezer at –80°C to determine enzyme activity. Furthermore, 0.5 g of fresh leaves were weighed and ground in 2 mL of buffers (10 µmol DDT, 0.5 mM EDTA, 10% glycerol (v/v), and 50 mM Tris-HCl (pH = 7.8) (Huang et al., 2021). After the grinding was centrifuged, a ribulose diphosphate carboxylase/oxygenase (Rubisco) activity assay kit (Solarbio Science & Technology Co., Ltd., Beijing, China) was used to assess Rubisco enzyme activity in the supernatant.

### 2.4 Chlorophyll content and dry matter accumulation

Three plants with measured gas exchange were selected during maize growth stages of V6, V12, R1, and R3 in two growing seasons and cut along the roots. All leaves were collected, and the leaf area of individual plants was determined using an LI-3100C leaf area meter (LI-COR, Biosciences, Lincoln, NE, USA). The leaf area index (LAI) was calculated by multiplying the leaf area of a single plant by the number of plants per unit area (Chen et al., 2022). Subsequently, 0.2 g of ear leaves were weighed and placed in 25 mL of anhydrous ethanol for 48 h with light-avoidance maceration. The absorbance of the extracts was measured at 665, 649, and 470 nm. The chlorophyll a (Chla), chlorophyll b (Chlb), and carotenoid (Car) contents were calculated according to Equations 1–3 (Liang et al., 2023). The total chlorophyll content (TCC) was measured by adding Chla and Chlb. The DMA per plant was assessed by placing the whole plant in an oven at 105°C for 30 min and drying at 85°C until constant weight was achieved. DMA per unit area was calculated by multiplying the dry weight per plant by the planting density (Li et al., 2024).


(1) $$Chl_{a}=13.95\times A_{665}-6.88\times A_{649}$$

(2) $$Chl_{b}=24.96\times A_{649}-7.32\times A_{665}$$

(3) $$\text{Car}=\frac{1000\times A_{470}-2.05\times Chl_{a}-114.8\times Chl_{b}}{245}$$

where, the $A_{665}$ , $A_{649}$ and $A_{470}$ are the absorbance of the extracts at 665, 649, and 470 nm, respectively.

### 2.5 Total soluble sugars, starch content, free amino acids, and soluble protein content

The R1 stage is critical for translocating assimilates, produced in the ear leaves, to the kernel and determining GY (Liu Z. et al., 2022; Rossini et al., 2023). We selected three plants with uniform growth at the R1 stage of the two maize growing seasons. Ear leaves were used to determine the TSS, SC, FAA, and SP. To evaluate TSS and SC, 0.5 g of fresh leaves were taken in 15 mL of distilled water. After a water bath in boiling water for 30 min, the solution was fixed to 100 mL. The TSS and SC were determined by the anthrone colorimetric method, as reported by Yue et al. (2022). Similarly, 0.5 g of fresh leaves were ground thoroughly in 0.2 M phosphate buffer (pH = 7.0). The FAA content was evaluated using the ninhydrin reagent method described by Zia et al. (2023). The SPC content in 0.2 g of fresh sample was determined using Coomassie brilliant blue G-250 reagent, as reported by Cong et al. (2023).

### 2.6 Key enzyme activities of sugar and N metabolism processes in ear leaves

At the R1 stage of the two maize growing seasons, three uniformly growing maize plants were selected from each plot, and fresh ear leaves were collected. The activities of invertase (INV), sucrose synthase (SS), sucrose phosphate synthase (SPS), nitrate reductase (NR), glutamine synthetase (GS), and glutamate synthetase (GOGAT) were measured using activity kits provided by Solarbio Science & Technology Co. Ltd. (Beijing, China). The tests were conducted according to the manufacturer’s instructions.

### 2.7 GY and N recovery efficiency

During the physiological maturity stage of the two maize growing seasons, a 10.0 m^2^ area from each plot was selected to harvest all ears, and the number of ears was recorded. Ears were threshed, dried (moisture content of grains not more than 14%), and weighed to calculate GY per unit area. Ten ears of uniform size were selected from the harvested ears and threshed to determine the thousand-grain weight. Six uniformly grown plants in each plot were selected to obtain the above-ground portion. The six plants were separated into stalks, leaves, bracteal leaves, and seeds, dried in an oven at 85°C to a constant weight, and weighed to record the DMA. The dried stalks, leaves (including bracteal leaves), and seeds were pulverized using a pulverizer (DFY500, Dingli Medical Equipment Co., Ltd, Wenzhou, China), sieved (0.5 mm mesh), desorbed using H_2_SO_4_-H_2_O_2_, and heated for digestion. The total N content in the stalks, leaves (including bracteal leaves), and seeds was determined using the Kjeldahl method (Bao, 2000). The plant nitrogen uptake (PNU, kg ha^–1^), NRE, %) and nitrogen agronomic efficiency (NAE, kg kg^–1^ N) were calculated according to Equations 4–6 reported by Wang et al. (2023).


(4) $$\text{PNU}=\sum \;{\text{PNC}}_{i}\times {\text{PDM}}_{i}/1000$$

(5) $$\text{NRE}=\frac{PNU_{f}-PNU_{0}}{N_{apl}}$$

(6) $$\text{NAE}=\frac{Y_{f}-Y_{0}}{N_{apl}}$$

PNC and PDM represent the plant N content (mg g^–1^) and plant dry matter (kg ha^–1^), respectively, and *i* denotes the stalks, leaves (including bracteal leaves), and seeds. $PNU_{f}$ and $PNU_{0}$ means the PNU of fertilization and non-fertilization treatments, respectively. $N_{apl}$ represents the total N application rate (kg N ha^–1^). $Y_{f}$ and $Y_{f}$ represent the GY (kg ha^–1^) of fertilization and non-fertilization treatments, respectively.

### 2.8 Statistical analysis

All data were analyzed using one-way analysis of variance (ANOVA) and the Statistical Package for the Social Sciences software (version 25.0; SPSS Inc., Chicago, USA). Duncan’s multiple range test was employed to compare significant differences between treatments (*p<* 0.05). The statistical data were visualized using Origin 2023 (Originlab, USA). Furthermore, we constructed structural equation models (SEM) and performed Mantel tests for sugar and N metabolites, sugar and N metabolism catalytic enzymes, photosynthesis processes, GY, and PNU using AMOS 26.0 (SPSS Inc., Chicago, USA) and the OmicShare platforms (https://www.omicshare.com/tools/). SEM fitness was determined by the chi-square degrees of freedom ratio (χ^2^/df ≤ 2.0), the goodness-of-fit index (> 0.90), and the root mean square error of approximation (≤ 0.1). The SEM was visualized using Microsoft PowerPoint 2019.

## 3 Results

### 3.1 LAI, Pn, and Rubisco activity

**Figure 2:**
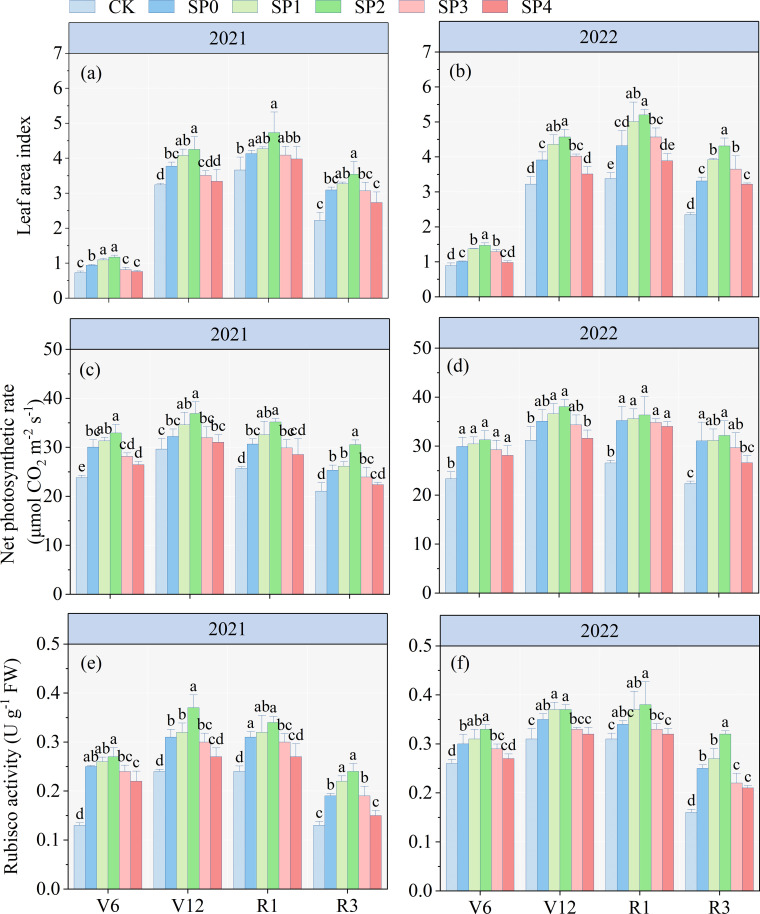
**(A–F)** Changes in leaf area index, net photosynthetic rate, and Rubisco activity under different treatments in 2021 and 2022. V6, V12, R1, and R3 correspond to the jointing, bell-mouth, silking, and milk maturity stages, respectively. CK refers to no chemical and organic nitrogen fertilizer. SP0 refers to 100% chemical nitrogen fertilizer (210 kg N·ha^–1^), and SP1, SP2, SP3, and SP4 refer to 15%, 30%, 45%, and 60% of chemical nitrogen fertilizer substituted with organic fertilizer, respectively. Different letters indicate significant differences at *p<* 0.05 by Duncan’s multiple range test.

The ANOVA results in **Table 2** revealed that LAI, Pn, and Rubisco activity at various growth stages significantly differed between the two growing seasons (*p<* 0.05). LAI, Pn, and Rubiso activity were significantly influenced by SP treatment (*p<* 0.05) and remained unaffected by year (Y) and SP interaction in most stages (*p* > 0.05). The LAI, Pn and Rubiso activity of all treatments initially increased and subsequently decreased with maize growth and development (**Figure 2**). Compared with the CK treatment, all SP treatments (SP0–SP4) increased maize LAI (**Figures 2A, B**), Pn (**Figures 2C, D**), and Rubiso activity (**Figures 2E, F**) at the V6, V12, R1, and R3 stages, highest increase was observed at the R1 stage. At the R1 stage, compared to the SP0 treatment, the SP1 and SP2 treatments increased LAI, Pn, and Rubiso activity by 9.7%–17.4% (*p<* 0.05), 3.7%–8.9%, and 6.0%–10.7% (*p<* 0.05), respectively. Conversely, the SP3 and SP4 treatments reduced LAI, Pn, and Rubiso activity by 2.5%–6.8%, 1.8%–5.1%, and 3.1%–9.4%, respectively. The LAI, Pn, and Rubisco activity of the SP4 treatment non-significantly differed from the CK treatment.

**Table 2:** The ANOVA of leaf area index (LAI), net photosynthetic rate (Pn), Rubisco activity, total chlorophyll content (TCC), carotenoid content (Car), dry matter accumulation (DMA), total soluble sugar content (TSS), starch content (SC), invertase activity (INV), sucrose synthase activity (SS), sucrose phosphate activity (SPS), free amino acids content (FAA), soluble proteins content (SP), nitrate reductase activity (NR), glutamine synthetase activity (GS), and glutamate synthetase activity (GOGAT).

Indicators	Stage	Source of variance		
		Y	SP	Y × SP
LAI	V6	221.9**	93.7**	11.4**
	V12	10.2**	25.5**	1.02ns
	R1	5.1*	14.0**	2.1ns
	R3	43.5**	41.2**	2.0ns
Pn	V6	0.01ns	26.2**	1.1ns
	V12	6.3*	9.4**	0.2ns
	R1	23.9**	15.6*	1.4ns
	R3	33.6**	16.4**	1.4ns
Rubisco activity	V6	214.0**	46.7**	8.7**
	V12	56.9**	27.9**	3.3*
	R1	33.1**	10.7**	0.6ns
	R3	148.4**	84.0**	3.5*
TCC	V6	4.3*	26.4**	1.0ns
	V12	45.7**	45.2**	1.9ns
	R1	1.3ns	17.9**	1.4ns
	R3	11.8**	45.0**	4.6*
Car	V6	4.6*	43.1**	0.3ns
	V12	0.1ns	22.4**	2.4ns
	R1	7.0*	40.1**	0.9ns
	R3	5.0*	22.1**	0.5ns
DMA	V6	107.0**	69.6**	1.7ns
	V12	4.1*	48.7**	5.6*
	R1	35.5**	77.6**	3.9*
	R3	2.0ns	21.3**	0.9ns
	Haverst	3.5*	18.5**	0.4ns
TSS	–	15.7**	133.6**	12.5**
SC	–	1712.7**	135.7**	1.7ns
FAA	–	168.0**	145.2**	6.9*
SP	–	14.7**	42.1**	1.3ns
INV	–	177.7**	201.0**	2.5ns
SS	–	58.9**	150.6**	0.9ns
SPS	–	323.8**	101.6**	2.1ns
NR	–	306.9**	84.4**	1.8ns
GS	–	2300.7**	276.2**	2.6ns
GOGAT	–	433.1**	194.1**	1.2ns

V6 refers to jointing stage, V12 refers to bell-mouth stage, R1 refers to silking, and R3 refers to milk maturity stages. Y refers to year, and SP refers to substituting partial chemical nitrogen fertilizers with organic fertilizers. Different letters indicate significant differences in the same year by Duncan’s multiple range test. *, p<0.05; **, p<0.01; ns, not significant.

### 3.2 Chlorophyll content and DMA

**Figure 3:**
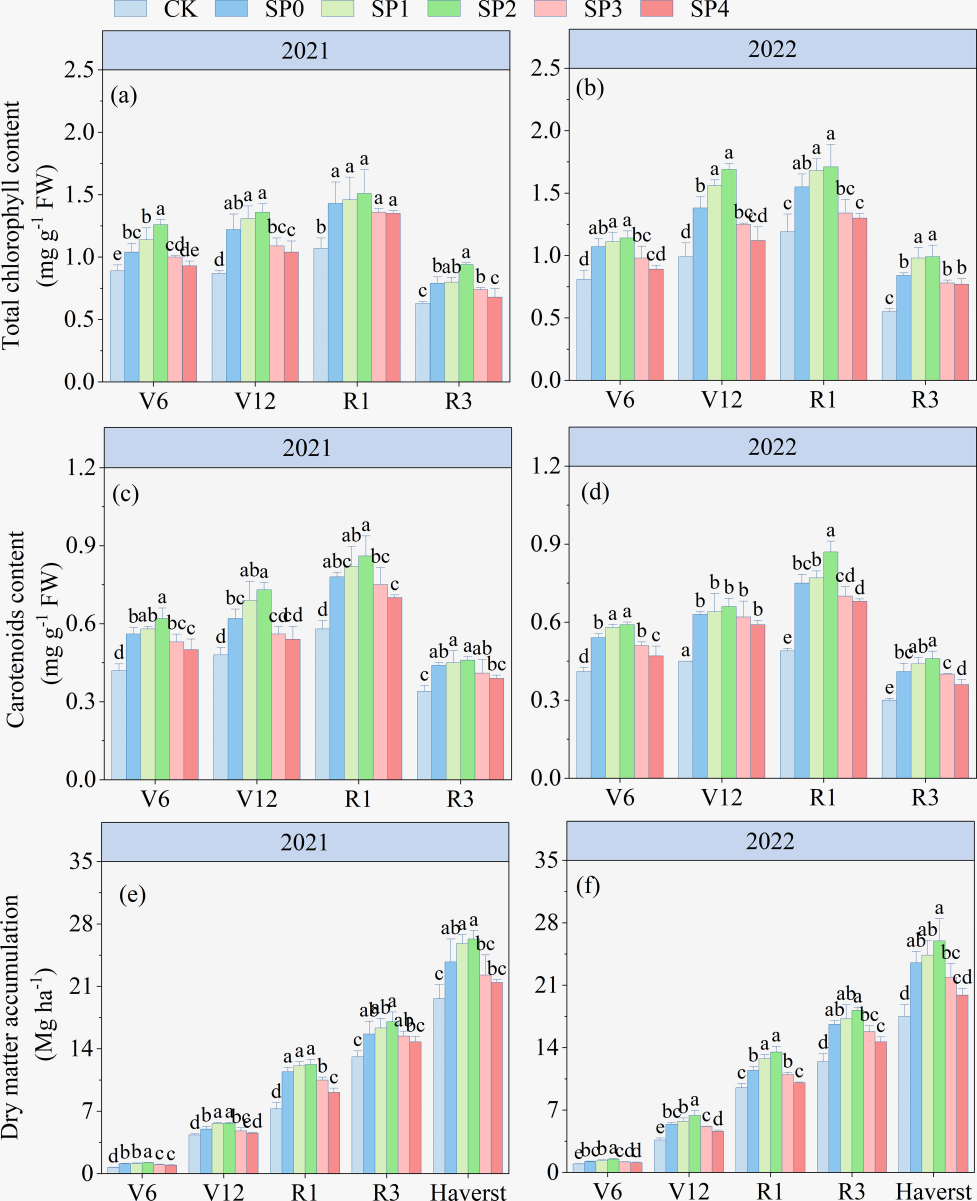
**(A–F)** Effect of different treatments on total chlorophyll content, carotenoid content, and dry matter accumulation at different growth stages of maize in 2021 and 2022. V6, V12, R1, and R3 correspond to the jointing, bell-mouth, silking, and milk maturity stages, respectively. CK refers to no chemical and organic nitrogen fertilizer. SP0 refers to 100% chemical nitrogen fertilizer (210 kg N·ha^–1^), and SP1, SP2, SP3, and SP4 refer to 15%, 30%, 45%, and 60% of chemical nitrogen fertilizer substituted with organic fertilizer, respectively. Different letters indicate significant differences at *p<* 0.05 by Duncan’s multiple range test.

The ANOVA results in **Table 2** demonstrated that TCC, Car contents, and DMA at various growth stages significantly differed between the two growing seasons (*p<* 0.05). TCC, Car contents, and DMA were significantly affected by SP treatment (*p<* 0.05), while they remained unaffected by Y and SP interaction in most stages (*p* > 0.05). The TCC and Car contents of ear leaves in all treatments increased and then decreased with maize growth and development, reaching the highest at the R1 stage. At the R1 stage, compared with the CK treatment, the TCC and Car contents of all SP treatments significantly increased by 17.7%–38.8% and 29.7%–49.3%, respectively (**Figures 3A, D**). Compared with the SP0 treatment, SP1–SP4 increased the TCC and Car contents in the ear leaves. The highest TCC and Car contents were recorded in the SP2 treatment, which significantly increased by 8.0% and 13.1% compared to the SP0 treatment. However, higher SP treatments (SP3 and SP4) decreased TCC and Car contents compared with the SP0 treatment. There was no significant difference in DMA between SP0, SP1, and SP2 treatments at maize harvest (**Figures 3E, F**). However, compared with the SP0 treatment, the SP4 treatment significantly reduced DMA by 12.6% (*p<* 0.05) by reducing the MDA_stalk_ (**Supplementary Table S1**; **Figures 3E, F**).

### 3.3 Soluble sugars, SC, FAA, and protein content

**Figure 4:**
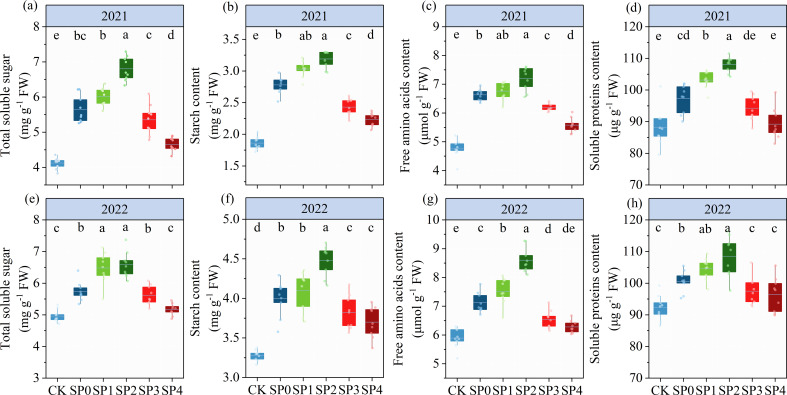
**(A–H)** Total soluble sugar, starch, free amino acids, and soluble protein content of different treatments at the silking stage (R1) in 2021 and 2022. CK refers to no chemical and organic nitrogen fertilizer. SP0 refers to 100% chemical nitrogen fertilizer (210 kg N·ha^–1^), and SP1, SP2, SP3, and SP4 refer to 15%, 30%, 45%, and 60% of chemical nitrogen fertilizer substituted with organic fertilizer, respectively. Different letters indicate significant differences at *p<* 0.05 by Duncan’s multiple range test.

As illustrated by the ANOVA results in **Table 2**, TSS, SC, FAA, and SP significantly differed between the two growing seasons (*p<* 0.05) and were significantly affected by SP treatment (*p<* 0.05). In addition, TSS and FAA were significantly influenced by the Y and SP interaction (*p<* 0.05). In both growing seasons, all SP treatments significantly increased TSS, SC, FAA, and SPC in ear leaves by 8.5%–38.3%, 16.3%–43.9%, 12.4%–35.0%, and 2.9%–15.2%, respectively, compared with the CK treatment (**Figure 4**). TSS, SC, FAA and SPC in ear leaves increased and then decreased with the increasing percentage of SP substituting chemical N fertilizer, reaching the highest in SP2 treatment and the lowest in the SP4 treatment. Compared with the SP0 treatment, the TSS, SC, FAA, and SPC increased in the SP1 and SP2 treatments by 9.7%–17.5%, 5.7%–13.4%, 3.8%–14.7%, and 5.1%–9.5%, respectively. However, aforementioned variables decreased in the SP3 and SP4 treatments by 3.7%–13.8%, 8.5%–13.7%, 7.5%–13.8%, and 3.2%–6.2%, respectively. The TSS, FAA, and SP contents in the SP4 treatment non-significantly differed from those in the CK treatment.

### 3.4 Enzyme activity of sugar metabolism in ear leaves

**Figure 5:**
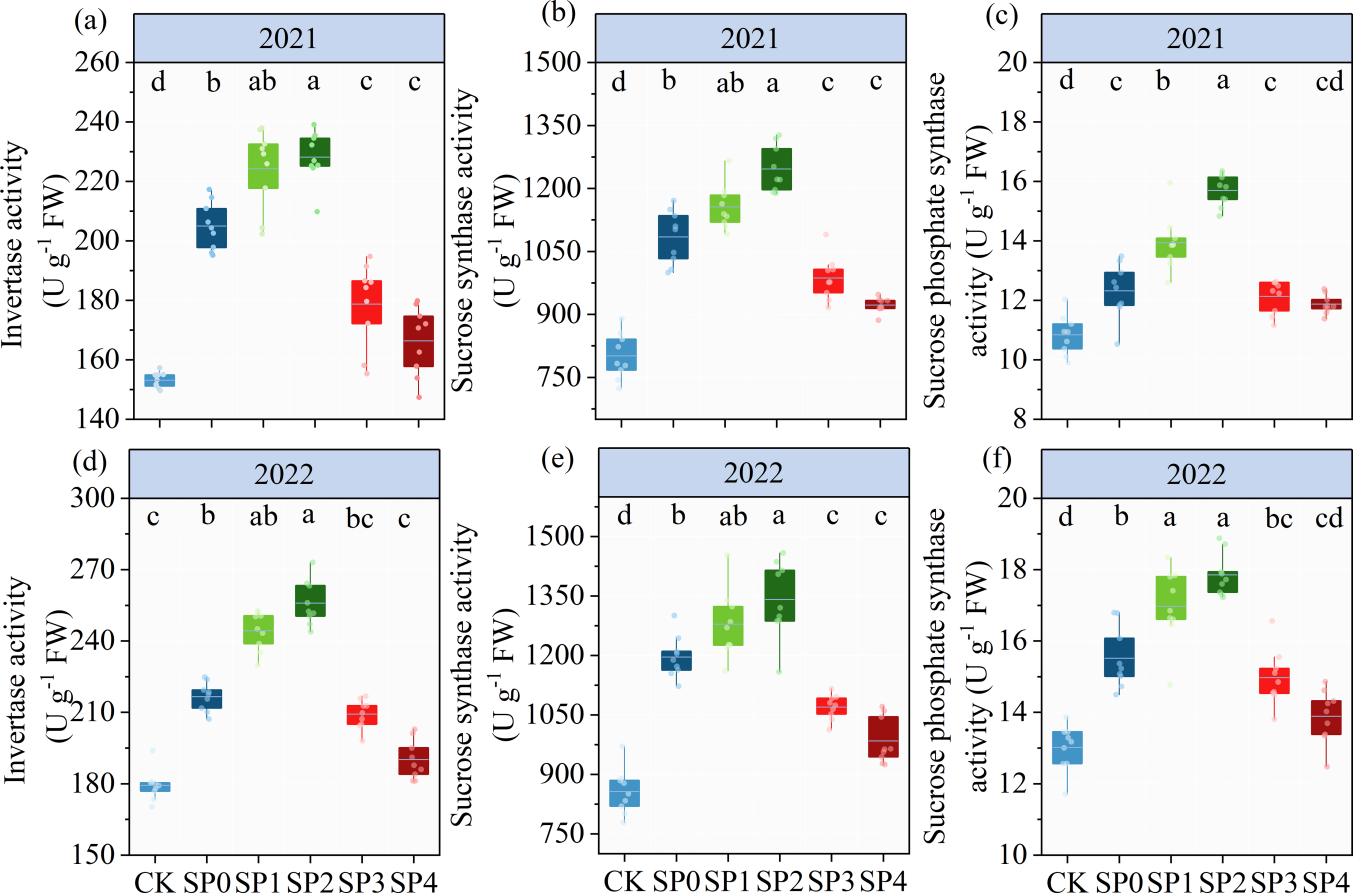
**(A–F)** The activities of invertase, sucrose synthase, and sucrose phosphate synthase under different treatments at the silking stage (R1) in 2021 and 2022. CK refers to no chemical and organic nitrogen fertilizer. SP0 refers to 100% chemical nitrogen fertilizer (210 kg N·ha^–1^), and SP1, SP2, SP3, and SP4 refer to 15%, 30%, 45%, and 60% of chemical nitrogen fertilizer substituted with organic fertilizer, respectively. Different letters indicate significant differences at *p<* 0.05 by Duncan’s multiple range test.

**Figure 6:**
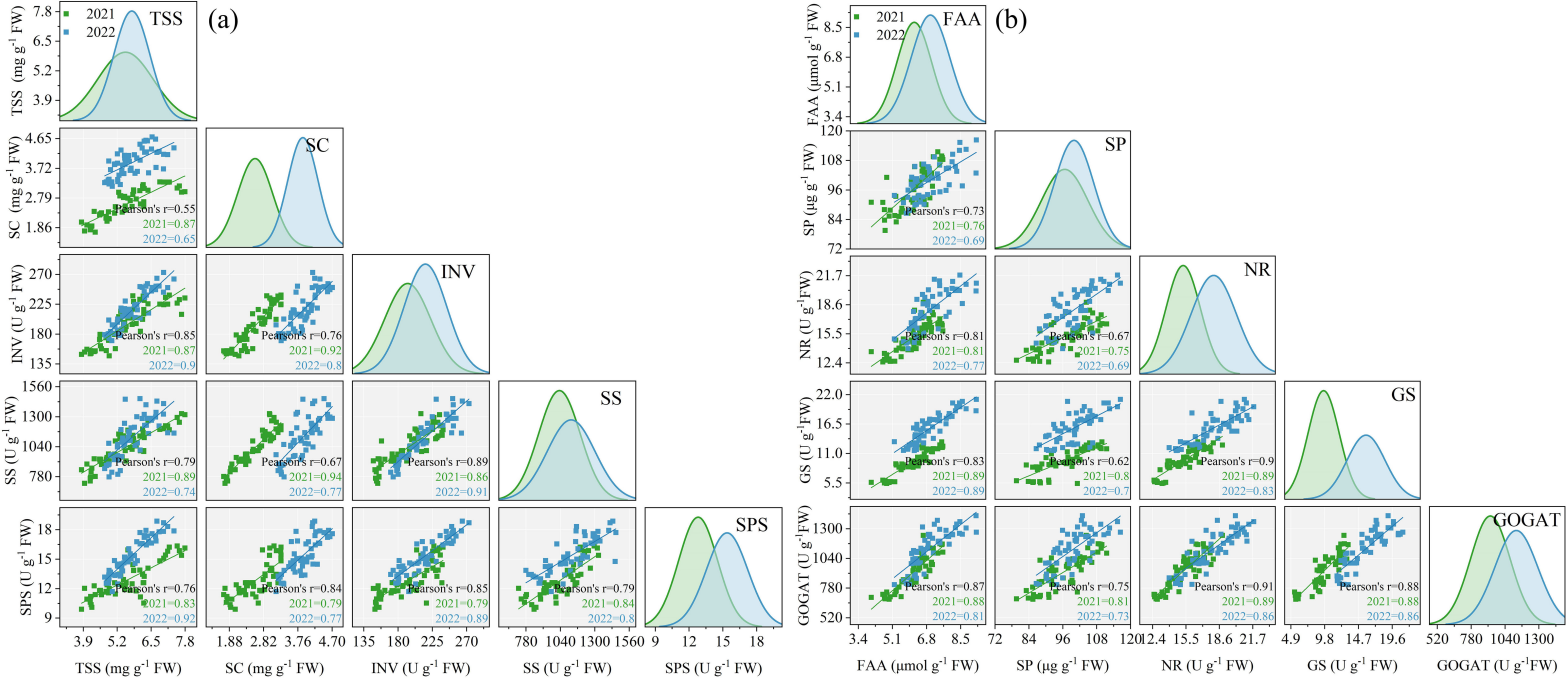
Correlation analysis between sugar metabolites and related enzyme activities **(A)** and nitrogen metabolites and related enzyme activities **(B)**. TSS, SC, INV, SS, and SPS refer to total soluble sugar content, starch content, invertase activity, sucrose phosphate activity, and sucrose phosphate activity, respectively. FAA, SP, NR, GS, and GOGAT refer to free amino acids content, soluble protein content, nitrate reductase activity, glutamine synthetase activity, and glutamate synthetase activity, respectively.

The ANOVA results in **Table 2** revealed that INV, SS, and SPS significantly differed between the two growing seasons (*p<* 0.05). INV, SS, and SPS were significantly impacted by SP treatment (*p<* 0.05) while remaining unaffected by Y and SP interaction (*p >* 0.05). In both growing seasons, the activities of INV, SS, and SPS in ear leaves significantly increased by 7.4%–41.4%, 15.0%–46.8%, and 8.1%–29.5%, respectively, in all SP treatments compared with the CK treatment (**Figure 5**). The activities of INV, SS, and SPS in ear leaves increased and then decreased with the increasing percentage of SP substituting chemical N fertilizer, reaching the highest in the SP2 treatment and the lowest in the SP4 treatment (**Figures 5A–F**). Compared with the SP0 treatment, the activities of INV, SS, and SPS significantly increased by 11.1%–14.7%, 6.8%–13.5%, and 11.3%–21.2%, respectively, in the SP1 and SP2 treatments, while they significantly decreased by 8.2%–15.6%, 9.9%–16.3%, and 2.5%–7.0% (*p* > 0.05), respectively, in the SP3 and SP4 treatments. The differences in SPS activities between the SP4 and CK treatments were non-significant. Correlation analysis found that the activities of INV, SS, and SPS were significantly positively correlated with TSS and SC contents in maize ear leaves in both growing seasons (**Figure 6A**).

### 3.5 Enzyme activity of N metabolism in ear leaves

**Figure 7:**
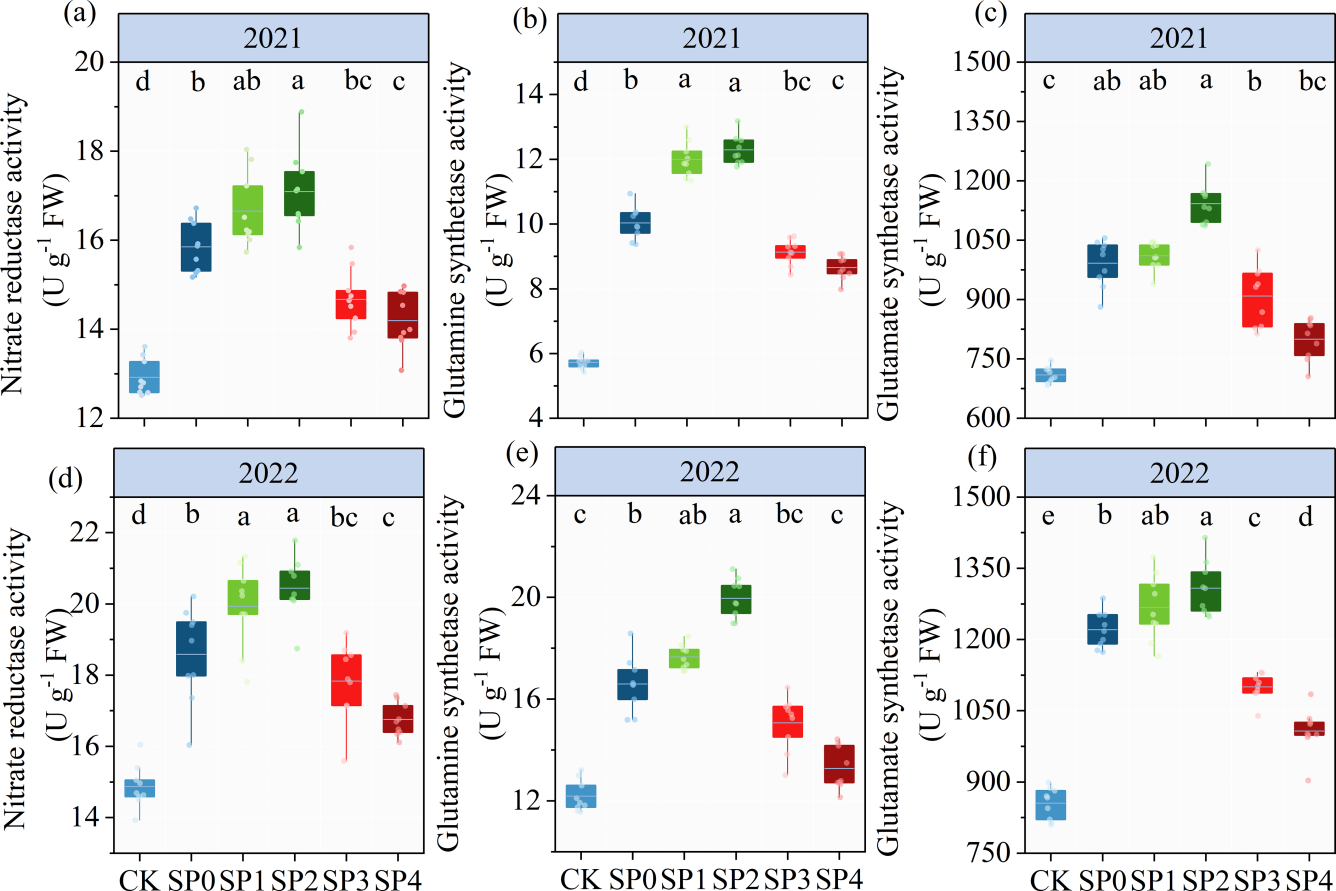
**(A–F)** The activities of nitrate reductase, glutamine synthetase, and glutamate synthetase under different treatments at the silking stage (R1) in 2021 and 2022. CK refers to no chemical and organic nitrogen fertilizer. SP0 refers to 100% chemical nitrogen fertilizer (210 kg N·ha^–1^), and SP1, SP2, SP3, and SP4 refer to 15%, 30%, 45%, and 60% of chemical nitrogen fertilizer substituted with organic fertilizer, respectively. Different letters indicate significant differences at *p<* 0.05 by Duncan’s multiple range test.

As represented in **Table 2**, ANOVA findings revealed that NR, GS, and GOGAT significantly differed between the two growing seasons (*p<* 0.05). NR, GS, and GOGAT were significantly influenced by the SP treatment (*p<* 0.05) while remaining unaffected by Y and SP interaction (*p* > 0.05). In both growing seasons, the activities of NR, GS, and GOGAT in ear leaves were significantly increased by 11.2%–31.4%, 30.1%–77.3%, and 15.3%–41.3%, respectively, in all SP treatments compared with the CK treatment (**Figure 7**). The activities of NR, GS, and GOGAT in ear leaves increased and then decreased with increasing percentage of SP substituting chemical N fertilizer, reaching the highest in the SP2 treatment and the lowest in the SP4 treatment (**Figures 7A–F**). Compared with the SP0 treatment, the activities of NR, GS, and GOGAT were significantly increased by 6.2%–8.9%, 13.0%–21.4%, and 2.9%–11.2%, respectively, in the SP1 and SP2 treatments, while they decreased by 5.7%–10.2%, 9.1%–16.9%, and 9.2%–18.4%, respectively, in SP3 and SP4 treatments. Correlation analysis revealed that NR, GS, and GOGAT activities in ear leaves were significantly positively correlated with FAA and SP contents in maize ears in both growing seasons (**Figure 6A**).

### 3.6 PNU, NU distribution, and NRE

**Figure 8:**
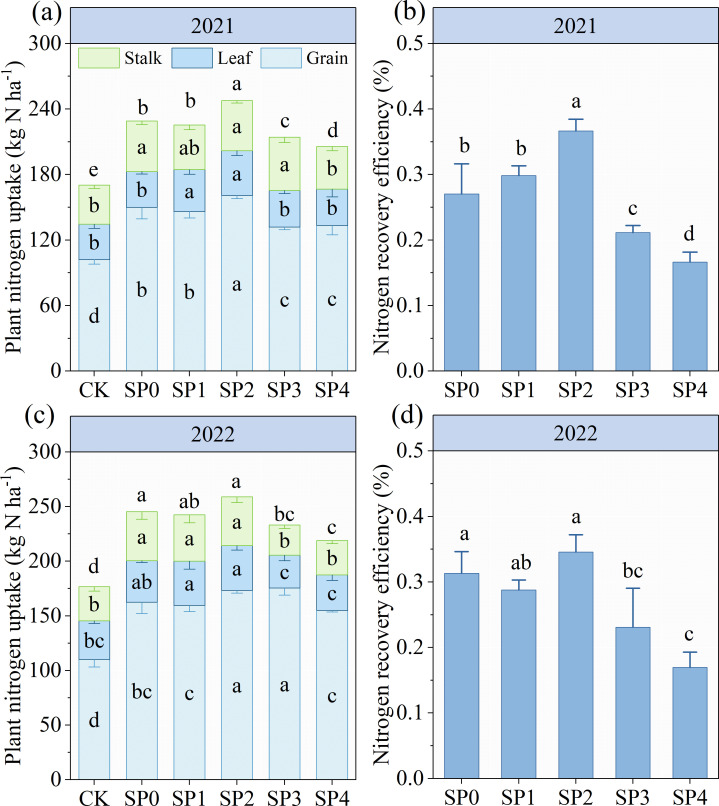
**(A–D)** The plant nitrogen uptake and recovery efficiency under different treatments at the harvest stage in 2021 and 2022. CK refers to no chemical and organic nitrogen fertilizer. SP0 refers to 100% chemical nitrogen fertilizer (210 kg N·ha^–1^), and SP1, SP2, SP3, and SP4 refer to 15%, 30%, 45%, and 60% of chemical nitrogen fertilizer substituted with organic fertilizer, respectively. Different letters indicate significant differences at *p<* 0.05 by Duncan’s multiple range test.

SP treatments (SP0–SP4) did not increase the total N content of stalk, leaf, and grain in two growing seasons compared with CK treatments (**Supplementary Table S1**). However, SP treatments significantly increased PNU by 20.1%–35.1% at harvest by increasing the DMA of stalk, leaf, and grain (**Figure 8**; **Supplementary Table S1**). The PNU increased and then decreased with increasing SP ratio; SP2 treatment exhibited the highest PNU. The PNU was 7.8% (*p*< 0.05 in 2021) higher in SP2 treatment compared with SP0 treatment in both growing seasons. However, SP3 and SP4 treatments reduced PNU by 6.6% (*p<* 0.05) and 12.2% (*p<* 0.05) by decreasing grains N uptake as compared with SP0 treatment (**Figure 8**). SP2 treatment increased NRE by 16.6%–111.9% (*p<* 0.05 in 2021) through increasing PNU compared with other treatments (**Figures 8B, D**).

### 3.7 Yield and yield components, NAE

The ear number (EN), kernel number (KN), thousand kernel weight (TKW), GY, and NAE of maize displayed no significant differences between the two growing seasons (**Table 3**). All SP treatments demonstrated no significant effect on EN; however, they significantly increased KN, TKW, and GY by 36.8%–45.5%, 10.4%–13.0%, and 41.2%–63.5%, respectively, as compared with the CK treatment. Additionally, the other SP treatments non-significantly affected KN and TKW compared with the SP0 treatment. The SP2 treatment increased GY by 9.2% (*p<* 0.05 in 2021) than the SP0 treatment by increasing KN per unit area (10.1%). The SP2 treatment resulted in the highest NAE in both growing seasons, significantly 27.8% higher than the SP0 treatment (**Table 3**).

**Table 3:** Ear number (EN), kernel number (KN), thousands kernel weight (TKW), grain yield (GY), and nitrogen agronomic efficiency (NAE) for different treatments in 2021 and 2022.

Year	Treatments	Ear number (Per m^–2^)	Kernel number (Per ear^–1^)	Kernel number (Per m^–2^)	Thousand kernel weight (g)	Grain yield (Mg ha^–1^)	NAE (kg kg^–1^ N)
2021	CK	5.4 ± 0.3b	405.6 ± 28.1b	2190.2 ± 34.3c	332.7 ± 2.8b	8.1 ± 0.2c	–
	SP0	5.7 ± 0.1ab	557.6 ± 10.6a	3178.3 ± 238.8b	371.7 ± 38.2a	12.4 ± 0.2b	20.5 ± 1.2c
	SP1	5.8 ± 0.1ab	565.4 ± 28.1a	3279.3 ± 281ab	373.6 ± 19.2a	12.7 ± 0.1ab	21.9 ± 0.6b
	SP2	6.0 ± 0.5a	594.2 ± 47.8a	3565.2 ± 153.5a	377.2 ± 27.6a	13.4 ± 0.4a	25.2 ± 2.1a
	SP3	5.7 ± 0.2ab	570.7 ± 8.1a	3253.1 ± 118.4ab	375.5 ± 42.3a	12.1 ± 1.5ab	22.9 ± 6.9b
	SP4	5.5 ± 0.2ab	572.3 ± 27.9a	3147.7 ± 128.3b	374.1 ± 6.1a	11.5 ± 0.4b	22.4 ± 1.9b
2022	CK	5.5 ± 0.2a	417.3 ± 24.2c	2295.2 ± 105.1c	341.5 ± 28.6c	8.6 ± 0.2c	–
	SP0	5.7 ± 0.7a	567.8 ± 24.5ab	3236.5 ± 268.7b	372.6 ± 31.9b	12.6 ± 0.5ab	19.0 ± 1.2bc
	SP1	5.7 ± 0.7a	576.4 ± 24a	3285.5 ± 267.5b	381.2 ± 9.1ab	13.2 ± 0.3ab	21.9 ± 1.3ab
	SP2	5.8 ± 0.2a	602.8 ± 41.7a	3496.2 ± 163.9a	384.5 ± 41.2a	13.9 ± 0.7a	25.2 ± 3.2a
	SP3	5.7 ± 0.2a	582.3 ± 17.2a	3319.1 ± 130ab	380.3 ± 14.6ab	13.4 ± 0.6ab	22.9 ± 1.2a
	SP4	5.7 ± 0.1a	580.1 ± 14.8a	3206.6 ± 276.2b	378.5 ± 39.6b	12.2 ± 0.8b	17.1 ± 0.8c
	Source of variance						
	Y	0.5ns	1.3ns	0.4ns	0.4ns	2.3ns	1.8ns
	SP	1.01ns	38.4**	33.5**	3.1*	80.6**	4.3*
	Y × SP	0.2ns	0.02ns	0.2ns	0.05ns	1.1ns	1.1ns

CK refers to no chemical and organic nitrogen fertilizer, SP0 refers to 100% chemical nitrogen fertilizer (210 kg N·ha^-1^), and SP1, SP2, SP3, and SP4 refers to 15%, 30%, 45%, and 60% of chemical nitrogen fertilizer substituted with organic fertilizer, respectively. Y refers to year, and SP refers to substituting partial chemical nitrogen fertilizers with organic fertilizers. Different letters indicate significant differences in the same year by Duncan’s multiple range test. *, p<0.05; **, p<0.01; ns, not significant.

### 3.8 Correlation analysis and SEM

**Figure 9:**
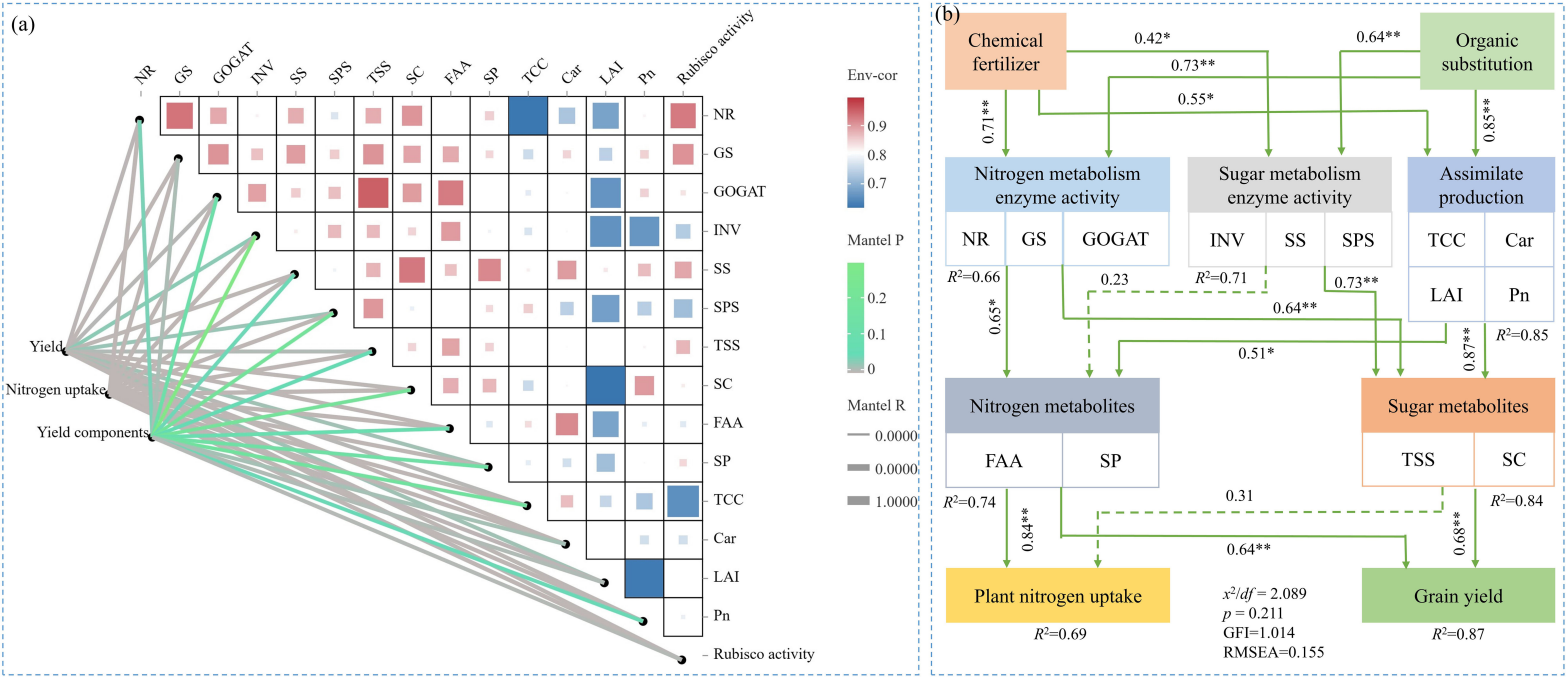
Correlation analysis between sugar metabolism processes, nitrogen metabolism processes, and photosynthesis processes in maize ear leaves, and Mantel test of these processes with grain yield and yield components **(A)**. Structural equation modeling (SEM) reveals the effects of carbon and nitrogen metabolic processes under different treatments on plant nitrogen uptake and grain yield **(B)**. Indicators that indicate the process of sugar metabolism include total soluble sugar content (TSS), starch content (SC), invertase activity (INV), sucrose synthase activity (SS), and sucrose phosphate activity (SPS). Indicators that indicate the process of nitrogen metabolism include free amino acids content (FAA), soluble proteins content (SP), nitrate reductase activity (NR), glutamine synthetase activity (GS), and glutamate synthetase activity (GOGAT). Indicators that indicate the process of photosynthesis processes include total chlorophyll content (TCC), carotenoid (Car), leaf area index (LAI), and net photosynthetic rate (Pn). Yield components included ear number, kernel number, and thousand kernel weight. χ2/df, GFI, and RMSEA refers to the chi-square degrees of freedom ratio (χ2/df ≤ 2.0), goodness-of-fit index (GFI > 0.90), and root mean squared error of approximation (RMSEA ≤ 0.1), respectively.

A positive correlation was observed between sugar metabolism (TSS, SC, INV, SS, and SPS), N metabolism (FAA, SP, NR, GS, and GOGAT), and photosynthesis (TCC, Car, LAI, Pn, and Rubisco activity) in maize ear leaves. The correlation coefficients among these indexes were all greater than 0.5 (**Figure 9A**). The Mantel test results revealed that sugar and N metabolism and photosynthesis were significantly positively correlated with maize GY, yield components, and PNU (*p<* 0.05) (**Figure 9B**). SEM indicated that SP indirectly increased carbon and N metabolites in ear leaves, PNU and GY at harvest via directly positively affecting enzyme activities related to sugar and N metabolism, and photosynthesis (**Figure 9B**).

## 4 Discussion

### 4.1 Suitable SP substitution ratio promoted plant growth and increased DMA by improving assimilate productivity in the canopy

The assimilate production capacity of the canopy is the foundation for DMA and crop yield formation. Maximizing the use of light energy and enhancing photosynthetic efficiency are the primary determinants for high crop yields (Guo et al., 2022). In this study, SP1 and SP2 treatments increased LAI and Pn by increasing chlorophyll content (TCC and Car) and Rubisco activity in leaves at different stages of maize, compared to the SP0 treatments (**Figures 2**, **3**). Increased LAI, chlorophyll content, Pn, and Rubisco activity at the maize vegetative growth stage (before R1) resulted in higher efficiency of light interception and assimilate production, and assimilates were stored in leaves and stalks, increasing DMA (Suárez et al., 2022). Similar results were obtained in our study (**Figure 3**). The SP1 and SP2 treatments maintained high chlorophyll content, Pn, and Rubisco activity in maize leaves at the reproductive growth stage, demonstrating a positive effect on the sustained source activity and transfer of leaf-produced assimilates to the grain (Ma et al., 2021; Zhai et al., 2024). Therefore, GY under SP1 and SP2 treatments was increased by 3.6%–9.2% at harvest compared to the SP0 treatment.

Furthermore, SP3 and SP4 treatments resulted in varying degrees of reduction in LAI, chlorophyll content, Pn, and Rubisco activity at different stages compared to the SP0 treatment. There were no significant differences in LAI, chlorophyll content, Pn, and Rubisco activity in the SP4 treatment compared to the CK treatment (**Figures 2**, **3**). Ma et al. (2024) and Fei (2024) reported that excessive SP reduced soil nutrient content, enzyme activity, and microbial community abundance, causing an insufficient supply of inorganic nitrogen to the soil, negatively impacting maize plant growth (He et al., 2022a; Zhai et al., 2022). Wu X. Y. et al. (2024) found that SP substitution above 25% resulted in an insufficient supply of fast-acting nutrients to maize during the vegetative growth stage, which decreased leaf growth and DMA by reducing chlorophyll content and Pn and negatively affecting the reproductive growth stage of maize. However, in this study, substitution of more than 30% negatively affected maize growth, which may be related to the different soil and climatic conditions in the experimental regions (Bhunia et al., 2021; Cao et al., 2023).

### 4.2 Suitable SP substitution ratio increased GY by promoting C/NM in ear leaves

This study found that SP1 and SP2 increased carbohydrate accumulation in leaves by increasing the photosynthetic productivity of the canopy compared with the SP0 treatment, resulting in an increase in TSS and SC in ear leaves by 9.7%–17.5% and 5.7%–13.4%, respectively. SP1 and SP2 also increased FAA and SPC in ear leaves by 3.8%–14.7% and 5.1%–9.5%, respectively, compared with the SP0 treatment (**Figure 4**). Suitable substitution ratios can maintain the fast-acting nutrient content of the soil at different growth stages of maize, increased inorganic N content of the soil positively effect on accelerating the enzymatic reactions related to C/NM (Muhammad et al., 2022; Yue et al., 2022; Ren et al., 2023). Correlation analyses and SEM results of this study revealed that the increased carbon and N metabolites were mainly associated with increased activities of enzymes involved in C/NM in the leaves, and these enzyme activities were directly, significantly, and positively regulated by the SP treatment (**Figures 6**, **9**). The carbon and N metabolites in the leaves transferred to the ear increased the number of grains in the ears and TKW at the harvest stage, increasing GY by 8.0% under the SP2 treatment compared to the SP0 treatment (**Table 2**). Therefore, the SP1 and SP2 promoted GY formation via increasing sugar and N metabolism processes in the ear leaves of maize during the reproductive growth stage.

### 4.3 Suitable SP substitution ratio improved NRE by regulating N supply

In this study, the PNU at harvest was 5.8% (two-year average, *p<* 0.05 in 2021) higher in the SP2 treatment compared to the SP0 treatment, while the SP4 treatment significantly reduced PNU (**Figure 8**). Our results were consistent with the findings of Wu X. Y. et al. (2024) and Lou et al. (2024). This could be because substituting a small amount of chemical N (30%) with SP ensured nutrient supply during maize’s early and middle stages. This allowed the plant to rapidly absorb available N from the soil and form a sufficiently large source (Liang et al., 2023). On the contrary, an excessive SP substitution rate can slow plant growth due to insufficient supply of fast-acting nutrients in the early stage, reducing N uptake and accumulation (Wang et al., 2023). The SP2 treatment in this study improved N uptake and utilization by synchronizing N demand for maize growth, resulting in the highest PNU (247.5 kg N ha^–1^), GY (13.7 Mg ha^–1^), NRE (36%), and NAE (25.2 kg kg^–1^) (**Figure 8**; **Table 3**).

Although increasing the rate of SP application can enhance soil structure and soil fertility, studies, including ours, found that SP is low in nutrients, challenging to transport and apply (high cost), and cannot be applied in large quantities to satisfy crop growth needs (Bhunia et al., 2021). Therefore, an excessive substitution rate of SP is not recommended. This study only revealed the effects of leaf sugar and N metabolism and assimilate productivity on yield formation at the ear leaves in the SP substituting chemical N treatments. However, the soil fertilizer supply capacity is related to soil physicochemical properties and microbial diversity (Gu et al., 2019; Poveda, 2021; Yan et al., 2023). In the future, we should focus on the regulation of organic fertilizer replacing partial chemical N fertilizers on soil physicochemical properties and microbial diversity in maize planting system to provide new perspectives on how SP replacing chemical N fertilizers can improve maize yield and N use efficiency.

## 5 Conclusion

In the rainfed maize cropping system, compared with the SP0 treatment, the SP2 treatment improved canopy assimilate production capacity and dry matter accumulation at harvest by increasing the leaf area index, pigment content, net photosynthetic rate, and Rubisco activity of ear leaves. Additionally, SP2 treatment increased sugar and N metabolites by accelerating the enzymatic reactions related to sugar and N metabolism in the ear leaves during the reproductive growth stage of maize. Increased N metabolites enhanced plant N uptake and N recovery efficiency, while increased sugar metabolites promoted seed formation and improved grain yield. However, excessive SP replacement negatively affected canopy assimilate production capacity, slowed plant growth during the vegetative growth stage, and reduced sugar and N metabolism during the reproductive growth stage, deterring grain yield formation and plant N uptake. Therefore, in rainfed maize cropping systems, it is recommended that 30% of chemical N fertilizers should be substituted with organic fertilizer to improve N use efficiency without reducing grain yield.

## Data Availability

The raw data supporting the conclusions of this article will be made available by the authors, without undue reservation.
