# Supplementary material for: Substituting partial chemical nitrogen fertilizers with organic fertilizers maintains grain yield and increases nitrogen use efficiency in maize
Source: Front Plant Sci. 2024 Sep 18;15:1442123. doi: 10.3389/fpls.2024.1442123 (PMC11446218; doi:10.3389/fpls.2024.1442123)
Supplement: Supplementary file 1 [file Table1.docx]

**Table S1** Dry matter accumulation (DMA) and total nitrogen content (NC) of maize at harvest in stalks, leaves and grains under different substituting partial chemical nitrogen fertilizers with organic fertilizers treatments.

| Year | Treatments | DMA_stalk_  Mg ha^-1^ | DMA_leaf_  Mg ha^-1^ | DMA_grain_  Mg ha^-1^ | NC_stalk_  mg g^-1^ | NC_leaf_  mg g^-1^ | NC_grain_  mg g^-1^ |
| --- | --- | --- | --- | --- | --- | --- | --- |
| 2021 | CK | 6.5±0.1c | 3.1±0.2c | 8.1±0.2c | 5.5±0.2ab | 10.5±0.4a | 12.6±0.7a |
|  | SP0 | 8.1±0.3ab | 3.6±0.1b | 12.4±0.2ab | 5.7±0.1ab | 9.1±0.6b | 12.1±0.2a |
|  | SP1 | 8.7±0.2ab | 3.7±0.1b | 12.7±0.1ab | 4.7±0.1c | 10.4±1.1a | 11.5±0.7ab |
|  | SP2 | 9.2±0.2a | 4.4±0.2a | 13.4±0.4a | 5.0±0.2b | 9.3±1.3ab | 12.0±0.7a |
|  | SP3 | 7.6±0.4bc | 3.5±0.2bc | 12.1±1.5ab | 6.4±0.2a | 9.6±0.6ab | 10.9±0.4b |
|  | SP4 | 6.6±0.1c | 3.4±0.1bc | 11.5±0.4b | 5.9±0.5ab | 9.8±1.0ab | 11.6±0.6ab |
| 2022 | CK | 4.6±0.4d | 3.0±0.4b | 8.6±0.2c | 6.8±0.2a | 11.8±0.1a | 12.8±0.4ab |
|  | SP0 | 7.6±0.4ab | 3.3±0.1b | 12.6±0.5ab | 5.9±0.4b | 11.5±0.8a | 12.9±0.3ab |
|  | SP1 | 7.2±0.4b | 4.0±0.1a | 13.2±0.3ab | 5.9±0.4b | 10.1±0.3ab | 12.1±0.5b |
|  | SP2 | 8.0±0.2a | 4.1±0.2a | 13.9±0.7a | 5.6±0.1b | 10.0±0.7ab | 13.9±1.0a |
|  | SP3 | 5.1±0.3cd | 3.3±0.2b | 13.4±0.6ab | 5.4±0.2b | 9.1±0.9b | 13.1±1.3ab |
|  | SP4 | 5.6±0.4c | 3.4±0.1b | 12.2±0.8b | 5.6±0.6b | 9.6±1.4b | 12.7±0.4b |
|  | Source of variance | | | | | | |
|  | Y | 199.6** | 13.9** | 2.3ns | 0.1ns | 4.1* | 5.7* |
|  | SP | 91.9** | 32.1** | 80.6** | 1.4ns | 2.0ns | 0.4ns |
|  | Y×SP | 11.5** | 0.5ns | 1.1ns | 15.1** | 2.2ns | 0.6ns |

Note: DMA refers to the dry matter accumulation; NC refers to the total nitrogen content; CK refers to no chemical and organic nitrogen fertilizer, SP0 refers to 100% chemical nitrogen fertilizer (210 kg N·ha^-1^), and SP1, SP2, SP3, and SP4 refers to 15%, 30%, 45%, and 60% of chemical nitrogen fertilizer substituted with organic fertilizer, respectively. Y refers to year, and SP refers to substituting partial chemical nitrogen fertilizers with organic fertilizers. Different letters indicate significant differences in the same year by Duncan's multiple range test. *, p<0.05; **, p<0.01; ns, not significant.
